# 16S rDNA droplet digital PCR for monitoring bacterial DNAemia in bloodstream infections

**DOI:** 10.1371/journal.pone.0224656

**Published:** 2019-11-13

**Authors:** Ingrid Ziegler, Sofia Lindström, Magdalena Källgren, Kristoffer Strålin, Paula Mölling

**Affiliations:** 1 Department of Infectious Diseases, Örebro University Hospital, Örebro, Sweden; 2 School of Health and Medical Sciences, Örebro University, Örebro, Sweden; 3 Department of Laboratory Medicine, Västerås Hospital, Västerås, Sweden; 4 Department of Laboratory Medicine, Karlskoga Hospital, Karlskoga, Sweden; 5 Department of Infectious Diseases, Karolinska University Hospital, Stockholm, Sweden; 6 Department of Medicine Huddinge, Karolinska Institutet, Stockholm, Sweden; 7 Department of Laboratory Medicine, Örebro University Hospital, Örebro, Sweden; Defense Threat Reduction Agency, UNITED STATES

## Abstract

Repeated quantitative measurement of bacterial DNA on whole blood has been shown to be a promising method for monitoring bloodstream infection (BSI) with selected bacterial species. To enable broad use of this method, we developed a quantitative droplet digital PCR (ddPCR) method for 16S rDNA. It was validated with species-specific ddPCRs for *Staphylococcus aureus* (*nuc*), *Streptococcus pneumoniae* (*lytA*), and *Escherichia coli* (*uidA*) on spiked whole blood samples and on repeated whole blood samples (days 0, 1–2, 3–4, 6–8, and 13–15) from 83 patients with BSI with these pathogens. In these patients, 16S rDNA and species-specific DNA were detected in 60% and 61%, respectively, at least at one time-point. The highest positivity rates were seen in *S*. *aureus* BSI, where 92% of the patients were 16S rDNA-positive and 85% *nuc-*positive. Quantitative 16S rDNA and species-specific DNA showed strong correlations in spiked samples (r = 0.98; *p* < 0.0001) and clinical samples (r = 0.84; *p* < 0.0001). Positivity for 16S rDNA was rapidly cleared in patients with *S*. *pneumoniae* and *E*. *coli* BSI, but more slowly and sometimes persisted, in those with *S*. *aureus* BSI. The initial 16S rDNA load was higher in BSI patients with sepsis (Sepsis-3 definition) than without sepsis (median 2.38 vs. 0 lg10 copies/mL; *p* = 0.031) and in non-survivors than in survivors (median 2.83 vs. 0 lg10 copies/mL; *p* = 0.006). 16S rDNA ddPCR appears to be a promising method for bacterial DNA monitoring during BSI. The clinical value of such monitoring should be further studied.

## Introduction

Bloodstream infections (BSI) frequently lead to sepsis, one of the most common causes of death worldwide [1]. Blood culture (BC) is the gold standard for identifying the pathogen in BSI, but the method is limited by low sensitivity and a long turn-around-time [2]. Molecular methods for identification of BSI pathogens have been developed in the last decades [3–6]. Apart from pathogen identification, some molecular methods such as quantitative PCR enable quantification of bacterial DNA. Our group [7] and others [8–11] have linked a high load of bacterial DNA in the bloodstream to severity of sepsis. A few other studies have shown that the dynamics of bacterial DNA load reflect the disease course and response to treatment [12–14]. Accordingly, it has been proposed that repeated quantitative measurement of bacterial DNA could be used to evaluate the appropriateness of antibiotic treatment [15]. However, these studies of DNA dynamics have focused on DNA from only one bacterial species at a time. To be clinically useful, a diagnostic tool for DNA dynamics should monitor DNA from many different bacterial species.

The gene encoding 16S ribosomal RNA (16S rDNA) is present in all bacteria, which makes it an interesting target for detecting and quantifying bacterial DNA. A potential limitation of quantitative 16S rDNA detection, however, is that the copy number of 16S rDNA genes per genome differ between bacterial species, whereas most species-specific genes occur in only one copy [16, 17]. The 16S rDNA copy number per genome for the three most prevalent pathogens in community-onset BSI, *Staphylococcus aureus*, *Streptococcus pneumoniae* and *Escherichia coli* [18, 19], has been estimated at five to six, four, and seven, respectively [16].

Digital PCR is a PCR technology for absolute DNA quantification without the need of external standard curves, developed from real-time PCR [20]. Droplet digital PCR (ddPCR) [21] is today the most widespread application of digital PCR and has demonstrated a greater quantification precision and a higher reproducibility compared to real-time PCR, with a comparable sensitivity [22].

The primary objective of this study was to develop a tool to consecutively measure bacterial DNA in blood during BSI. For this purpose, we optimized a 16S rDNA ddPCR and validated it against species-specific ddPCRs for *S*. *aureus*, *S*. *pneumoniae*, and *E coli*. The secondary objective was to relate the 16S rDNA load to disease severity in BSI patients, hypothesizing that consecutively measuring 16S rDNA load would be clinically valuable.

## Materials and methods

### Patients

Patients who sought medical care at the Emergency Department at Örebro University Hospital, Örebro, Sweden, from February 2011 to June 2014, with suspected bacterial infection were eligible for enrollment in a prospective study, as described earlier [23]. Exclusion criteria were age under 18 and infection with human immunodeficiency virus (HIV), Hepatitis B, or C. All patients provided written informed consent. Together with BC bottles, EDTA blood samples were collected. When a BC bottle signaled positive and *S*. *aureus*, *S*. *pneumoniae*, or *E*.*coli* was identified (days 1–2), the patient was enrolled. Further blood samples, including EDTA tubes, were then collected on days 1–2, 3–4, 6–8, and 13–15.

### Blood cultures

According to routine practice, two pairs of BCs were collected per patient. For each BC a volume of 8–10 mL of venous blood was inoculated in a Bactec Aerobic/F bottle and the same volume in a BactecPlus Anaerobic/F bottle. The BC bottles were then incubated in the Bactec (Becton Dickinson and Company, Franklin Lakes, NJ, USA) system for up to seven days.

### Serial dilutions of samples with reference strain bacteria

Reference strain bacteria, *S*. *aureus* (CCUG 35601), *S*. *pneumoniae* (CCUG 33638), and *E*. *coli* (CCUG 7620), were cultivated overnight, and two to three colonies of each were mixed with 1 mL saline solution. Serial dilutions were then performed, first taking 2.5μL of the aliquot suspension into 2.5 mL of whole blood, for a dilution of 1:10^3^, then by diluting another five times down to 1:10^8^. These dilutions were later used in the species-specific ddPCRs, as positive controls in optimization and patient sample experiments and for validation of the ddPCR assays on bacteria-spiked whole blood. DNA concentrations of the different dilutions can be found in the result section.

A reference strain of *Haemophilus influenzae* (CCUG 33391), was prepared in the same way but only in dilutions of 1:10^3^ (7492 copies/mL) and 1:10^4^, for use as positive controls in all 16S rDNA ddPCR experiments.

### DNA extraction

Patient samples were frozen in aliquots of 1 mL EDTA blood at −70°C with 20% glycerol within 3 days of sample collection. Bacterial DNA was extracted using the Select NA Blood Pathogen Kit (Molzym, Bremen, Germany) on an Arrow instrument (Diasorin, Solna, Sweden) following the manufacturer’s instructions, and frozen. DNA extractions of the bacteria-spiked blood samples were performed in the same way, but those samples were not frozen as the PCR was run on the same day.

### Droplet digital PCR

In ddPCR, the PCR master mix, including the target template, is divided into about 15,000 water-in-oil droplets, and individual PCR reactions are performed in each of them. Following PCR, each droplet is analyzed to determine the fraction of PCR positive droplets in the original sample, after which the absolute target DNA template concentration can be calculated.

In the study, DNA was detected and quantified using the QX100 Droplet Digital PCR system (Bio-Rad Laboratories Inc., Pleasanton, CA, USA) according to the manufacturer’s instructions [24]. Template DNA (5μL), 1 x Bio-Rad Residual DNA Quantification Supermix, forward primer, reverse primer, probe and UV-radiated water was mixed to a final volume of 20μL, with an estimated DNA concentration range of 1–120000 copies per 20 μL reaction. The PCR mix was put into a droplet generator cartridge, droplet generator oil was added, and the cartridge was placed into a droplet generator. The generated droplet emulsion was transferred to a 96-well PCR plate and amplified in a thermal cycler. After amplification the plates were transferred to, and read, in a droplet reader.

For broad-range ddPCR, we used primers and a probe targeting the pan-bacterial 16S rDNA, as previously described [25]. For species-specific ddPCR, we used primers and probes targeting the *nuc* gene [26, 27], *lytA* gene [28], and *uidA* gene [29], all well-studied single-copy genes, specific for *S*. *aureus*, *S*. *pneumoniae*, and *E*. *coli*, respectively. All primer and probe sequences are presented in Table 1.

**Table 1:** Primer and probe sequences for 16S rDNA and species-specific *Staphylococcus aureus* (*nuc*), *Streptococcus pneumoniae* (*lytA*), and *Escherichia coli* (*uidA*) droplet digital PCRs.

Primer/probe^a^	DNA sequence 5´to 3´^b^
16S rDNA F	AACAGGATTAGATACCCTGGTAG
16S rDNA R	GGTTCTKCGCGTTGCWTC
16S rDNA P	6-FAM AACACTGCTCCACCGCT- MGBNFQ
*nuc* F	GCGATTGATGGTGATACGGTT
*nuc* R	AGCCAAGCCTTGACGAACTAAAGC
*nuc* P	6-FAM-ATGGTAGARAATGC-MGBNFQ
*lytA* F	CAGCGGTTGAACTGATTGA
l*ytA* R	TGGTTGGTTATTCGTGCAA
*lytA* P	6-FAM-AGCTGGAATTAAAACGCACGAG-MGBNFQ
*uidA* F	CAGTCTGGATCGCGAAAACTG
*uidA* R	ACCAGACGTTGCCCACATAATT
*uidA* P	6-FAM-ATTGAKCAGCRTTGG-MGBNFQ

^a^F = forward, R = reverse, P = probe

^b^6-FAM = 6-carboxyfluorescein, MGBNFQ = Minor groove binder non fluorescent quencher. K = G/T, R = A/G, W = A/T (wobble bases)

The design of the ddPCR assays included an optimization process, with protocols for adjustment of different parameters, in order to obtain ddPCR results with coherent clusters of positive droplets, well separated from the negative background. First, a temperature optimization was done where a temperature gradient from 55 to 65 ^0^C was used according to the manufacturer’s recommended thermal cycling protocol. The optimal annealing/extension temperature was set at 59°C for the 16S rDNA and *lytA* ddPCRs, 58°C for the *nuc* ddPCR, and 56°C for the *uidA* ddPCR. Thereafter experiments for primer and probe concentration titration were performed, where different concentrations of primers (0.5, 0.9 and 1.2 μM) and probes (0.1, 0.25, 0.4 μM) were tested. Optimal primer concentrations were set at 1.2 μM for both forward and reverse primers for the 16S rDNA, and 0.9μM for all species-specific genes. Optimal probe concentrations were set at 0.4 μM for the 16S rDNA PCR, 0.1 μM for the *nuc* and *lytA* PCRs, and 0.25 μM for the *uidA* PCR.

Duplicate DNA extractions from the whole blood dilutions of reference strain bacteria (*S*. *aureus*/CCUG 35601, *S*. *pneumonia*/CCUG 33638, *E*. *coli*/CCUG 7620 and *H*. *influenzae*/CCUG 33391) were used for the ddPCR optimization, in the dilution of 1:10^3^ in the temperature optimization, and 1:10^3^ and 1:10^4^ in primer and probe concentration experiments. UV-irradiated water was used as negative controls.

After ddPCR optimization, ddPCR was run on the whole blood dilutions (1:103–1:10^8^) of reference strain bacteria and on patient samples. All ddPCR experiments were performed in duplicate and the mean result reported. Duplicate DNA extractions from the whole blood dilutions of reference strain bacteria, in the dilution of 1:10^3^, were used as positive controls in the analyses on patient samples. The negative controls were presumed negative blood samples from 20 patients with negative BC and a laboratory confirmed non-bacterial diagnosis. A cut-off of 200 copies/mL was set for 16S rDNA ddPCR based on the results (mean plus two standard deviations) of the presumed negative samples.

### Clinical data and definitions

Clinical assessments were made on sampling days, and data were collected on demographics, co-morbidities, site(s) of infection, antibiotic treatment, length of hospital stay, intensive care unit admission, and mortality. Co-morbidity was evaluated using the Charlson Comorbidity Index [30]. Clinical conditions were classified according to the Sepsis-3 criteria, and patients with an acute change of ≥2 points in SOFA score due to the infection were considered to have sepsis [31].

### Statistics

Descriptive statistics are presented as medians with minimum and maximum values for continuous variables and as percentages for categorical variables. The non-parametric Mann-Whitney-U test was used for between-group comparisons, and Fischer’s exact test was used to compare proportions. A *p*-value of <0.05 was considered significant. The non-parametric Spearman’s rho test was used to assess correlation between the two different methods. Significant correlation was set at 0.01 (two-tailed). The SPSS software package (IBM corp., Armonk; NY, USA), version 22, was used for the statistical analyses. To calculate DNA template concentration in ddPCR, Poisson distribution statistics were performed using the Quantasoft software package (Bio-Rad Laboratories Inc., Pleasanton, CA, USA), version 1.4.

### Ethics

The Regional Ethical Review Board in Uppsala, Sweden (ref: 2009/024) approved the study.

## Results

### Patient characteristics

A total of 83 patients with BSI were enrolled in the study, 27 with *S*. *aureus* BSI, 30 with *S*. *pneumonie* BSI, and 26 with *E*. *coli* BSI. The patients were sampled repeatedly from admission (day 0) to day 13–15 (Table 2).

**Table 2:** Samples collected and analyzed with 16S rDNA ddPCR and species-specific ddPCR in 83 patients with *Staphylococcus aureus*, *Streptococcus pneumoniae*, or *Escherichia coli* bloodstream infection.

Results	Numbers (% of analyzed samples)				
	Day 0	Days 1–2	Days 3–4	Days 6–8	Days 13–15
**All patients (n = 83)**					
Samples collected	30	72	55	65	56
Samples analyzed	30	63	35	21	8
16S rDNA ddPCR +	18 (60)	35 (56)	16 (46)	8 (38)	4 (50)
Species-specific ddPCR +	21 (70)	34 (54)	16 (46)	4 (19)	0
***S*. *aureus* BSI patients (n = 27)**					
Samples collected	7	24	20	24	17
Samples analyzed	7	24	18	13	6
16s rDNA ddPCR +	7 (100)	18 (75)	13 (72)	8 (62)	4 (67)
*nuc* ddPCR +	7 (100)	19 (79)	10 (56)	3 (23)	0
***S*. *pneumoniae* BSI patients (n = 30)**					
Samples collected	9	28	15	25	25
Samples analyzed	9	28	7	3	1
16S rDNA ddPCR +	4 (44)	11 (39)	2 (29)	0	0
*lytA* ddPCR +	5 (56)	9 (32)	0	1 (33)	0
***E*. *coli* BSI patients (n = 26)**					
Samples collected	14	20	20	16	14
Samples analyzed	14	11	10	5	1
16S rDNA ddPCR +	7 (50)	4 (36)	2 (20)	0	0
*uidA* ddPCR +	9 (64)	6 (55)	6 (60)	0	0

As shown, not all patients had the full sample series, and not all samples were analyzed with ddPCR. The reason a sample was not analyzed was usually ddPCR negativity in the previous sample, or, in a few cases, lack of enough blood volume.

Demographic and clinical characteristics are given in Table 3.

**Table 3:** Demographic and clinical characteristics of the study-population.

	Total cohort^a^ N = 83	Bloodstream infection etiology^a^		
		*Staphylococcus aureus* n = 27	*Streptococcus pneumoniae* n = 30	*Escherichia coli* n = 26
***Demographic data***				
Median age, years	72 (24–93)	77 (24–93)	70 (30–89)	76 (29–93)
Female	39 (47)	4(15)	21 (70)	14 (54)
***Co-morbidities***				
Ischemic heart disease	24 (29)	9 (33)	6 (20)	9 (35)
COPD	6 (7)	1 (4)	5 (17)	0
Diabetes mellitus	18 (22)	6 (22)	2 (7)	10 (39)
Renal failure	7 (8)	3 (11)	0	3 (12)
Active malignancy	9 (11)	3 (11)	3 (10)	3 (12)
Charlson’s score	1 (0–8)	1 (0–8)	1 (0–8)	2 (0–7)
Immuno-suppressive treatment^b^	8 (10)	1 (4)	3 (10)	4 (15)
***Clinical data***				
Antibiotics before analyses	6 (7)	1 (4)	0	5 (19)
Adequate initial antibiotic treatment	80 (96)	25 (93)	30 (100)	25 (96)
Body temperature,°C, at admission	38.7 (35.6–41.0)	38.6 (35.6–40.9)	38.6 (35.8–41)	39.1 (37.1–40.0)
SOFA score increase at admission	1 (0–7)	1 (0–7)	2 (0–6)	1 (0–7)
Sepsis (SOFA score increase ≥2)	41 (49)	13(48)	22 (73)	6 (23)
Hospitalization length, days	8 (2–120)	14 (3–120)	5 (2–70)	4 (2–25)
Intensive care unit admission	16 (19)	7 (26)	7 (23)	2 (8)
90-days mortality	10 (12)	7 (26)	1 (3)	2 (8)
***Focus of infection***				
Endovascular	7 (8)	6 (22)	0	1 (4)
Skin/soft tissue/bone	16 (19)	15 (55)	1 (3)	0
Respiratory tract	26 (31)	1 (4)	25 (83)	0
Gastrointestinal	1 (1)	0	0	1 (4)
Urinary tract	21 (25)	0	0	21 (81)
Central nervous System	4 (5)	0	4 (13)	0
Unknown	8 (10)	5 (19)	0	3 (12)

^a^ Data are presented as numbers (percentages) for categorical variables and as median values (ranges) for continuous variables.

^b^Methotrexate, chemotherapeutics, or cortisol dosing equivalent to prednisolone ≥ 20mg.

The group with *S*. *pneumoniae* BSI had the most severe presentation at admission, with 73% meeting the Sepsis-3 criteria versus 48% in the *S*. *aureus* BSI group and 23% in the *E*. *coli* BSI group. However, the 90-day mortality rate was the lowest (3%) among patients with *S*. *pneumoniae* BSI compared with 26% and 8% in the *S*. *aureus* and *E*. *coli* BSI groups, respectively.

### 16S rDNA ddPCR results of spiked blood samples

Table 4 shows the results of the 16S rDNA ddPCR analyses compared with species-specific ddPCRs in serial dilutions of whole blood spiked with *S*. *aureus*, *S*. *pneumoniae*, or *E*. *coli*.

**Table 4:** Results from 16s rDNA and species-specific ddPCR analyses of serial dilutions of whole blood, spiked with *Staphylococcus aureus*, *Streptococcus pneumoniae* and *Escherichia coli*.

Dilution factor	*S*. *aureus*		*S*. *pneumoniae*		*E*. *coli*	
	16S rDNA copies/mL	*nuc* DNA copies/mL	16S rDNA copies/mL	*lytA* DNA copies/mL	16S rDNA copies/mL	*uidA* DNA copies/mL
10^3^	1,127,502	229,000	448,500	112,150	2,075,003	314,500
10^4^	115,500	22,500	42,000	9,775	190,750	23,050
10^5^	26,574	3,475	6,152	1,600	38,003	4,950
10^6^	4,950	235	2,024	143	5,950	775
10^7^	1,452	180	352	60	1,750	300
10^8^	402	0	452	0	287	0

**Fig 1:**
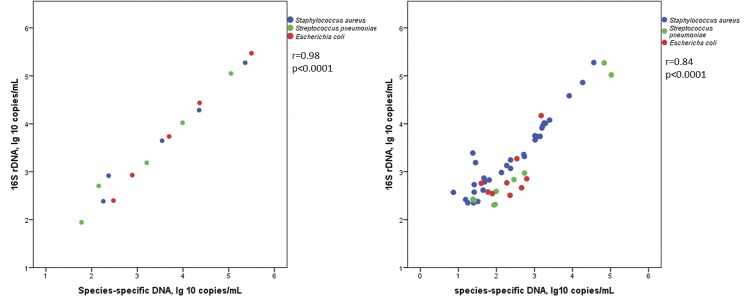
16S rDNA in relation to species-specific DNA (*nuc* for *Staphylococcus aureus*, *lytA* for *Streptococcus pneumoniae*, and *uidA* for *Escherichia coli*), in samples with DNA detected by both methods. (A) Whole blood samples spiked with bacteria in serial dilutions; (B) Whole blood samples from patients sampled repeatedly during bloodstream infection, one to four samples per patient.

For all bacteria, the last dilution with a positive result for 16S rDNA was 1:10^8^, and for species-specific DNA it was 1:10^7^. 16S rDNA was still detected in the dilution with the lowest concentration, indicating an at least 10-fold lower limit of detection for the 16S rDNA ddPCR than for the species-specific ddPCRs. The correlation between 16S rDNA and species-specific DNA for all spiked samples, positive in both methods, is illustrated in Fig 1A.

The median ratio between the 16S rDNA and the species-specific load was 6.6 (range, 3.9–21.1), for all samples together, 7.6 (range, 4.9–21.1) for *S*. *aureus*, 4.3 (range, 3.9–14.1) for *S*. *pneumoniae*, and 7.7 (range, 5.8–8.3) for *E*. *coli*. There was no significant difference in median ratios between the three bacteria groups (*p* = 0.22, 0.22, and 0.84 for *S*. *aureus* vs. *S*. *pneumoniae*, *S*. *aureus* vs. *E*. *coli*, and *S*. *pneumoniae* vs. *E*. *coli*, respectively). In a closer look at the results from the three dilutions with the highest bacteria concentration, the median ratios were nearer to the assumed 16S rDNA copies/genome for each pathogen: 5.1 (range: 4.9–7.7) for *S*. *aureus*, 4.0 (range, 3.9–4.3), for *S*. *pneumoniae*, and 7.6 (range: 6.6–8.3) for *E*. *coli*.

### 16S rDNA positivity in BSI patients

Sample frequency and positivity rates for 16S rDNA compared with species-specific ddPCR in blood from patients with BSI caused by *S*. *aureus*, *S*. *pneumoniae*, and *E*. *coli* are summarized in Table 2. The highest positivity rates were seen in *S*. *aureus* BSI, where 92% of the patients were 16S rDNA-positive and 85% *nuc-*positive, at least at one time point. Corresponding figures for *E*. *coli* and *S*. *pneumoniae* BSI were 46% (16S rDNA)/62% (*uidA*), and 43% (16S rDNA)/40% (*lytA*), respectively. Among patients with *S*.*aureus* BSI, both methods had a sensitivity of 100% on admission (day 0), and bacterial DNA was still detected in blood by both methods in about 80% of cases on days 1–2. Thereafter, positivity rates declined, and on days 13–15 no patients had detectable *nuc* DNA, while four still had detectable 16S rDNA in blood. Initial sensitivity was lower among patients with *S*. *pneumoniae* and *E*. *coli* BSI, and only a few patients had detectable bacterial DNA with any of the methods on days 3–4.

### Dynamics of bacterial DNA loads during the course of BSI

**Fig 2:**
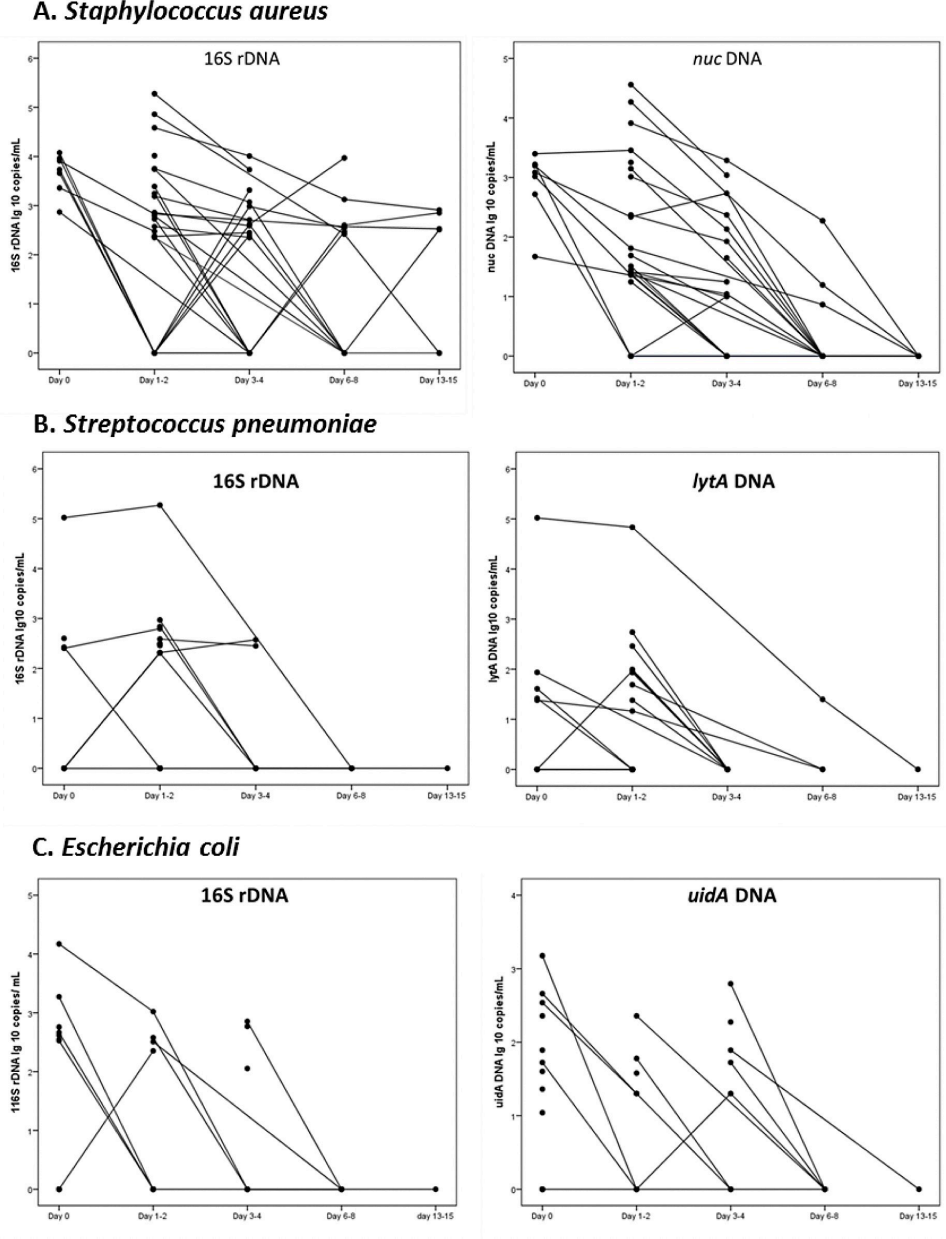
Quantitative data of 16S rDNA and species-specific DNA. (A) *nuc* for *Staphylococcus aureus*, (B) *lytA* for *Streptococcus pneumoniae*, and (C) *uidA* for *Escherichia coli* in individual patients with bloodstream infection and detected DNA.

Fig 2 demonstrates the dynamics of the bacterial DNA load in whole blood in the groups with *S*. *aureus* (A), *S*. *pneumoniae* (B), and *E*. *coli* (C) BSI measured with 16S rDNA and species-specific ddPCR.

The results from the *S*. *aureus* species-specific ddPCR have recently been published in a separate article focusing on *nuc* DNA loads in *S*. *aureus* bacteremia [32].

Results from the 16S rDNA and the species-specific ddPCR for cases shown positive by both methods were significantly correlated for *S*. *aureus* (r = 0.92, *p* < 0.0001) and *S*. *pneumoniae* (r = 0.83, *p* = 0.005), but not for *E*. *coli* (r = 0.57, *p* = 0.11). In the entire study group there was a significant correlation between results for the two methods (Fig 1B). Among patients with positive ddPCR in both methods, the median 16S rDNA/species-specific DNA ratio was 5.4 (range: 1.0–102.1). In all six cases where the ratio was higher than 15, the species-specific DNA load was low, at a median of 25 (range: 7–47) copies/mL. The median 16S rDNA/species-specific DNA ratios for the different BSI species were 6.5 (range: 3.8–102.1) for *S*. *aureus*, 2.5 (range: 1.0–11.1) for *S*. *pneumoniae*, and 4.5 (range: 1.0–14.4) for *E*. *coli*. There was a significant difference in median ratio between the *S*. *aureus* and *S*. *pneumoniae* groups (*p* = 0.002) but not between the *S*. *aureus* and *E*.*coli* groups (*p* = 0.66) or between the *S*. *pneumoniae* and *E*.*coli* groups (*p* = 0.052).

In most cases with repeatedly positive ddPCR, the load gradually fell until it was negative, but in a few cases the load increased between two sampling points (Fig 2). Four *S*. *aureus* cases were still positive for 16S rDNA at days 13–15. Three of them had stable 16S rDNA compared to the previous assessment point (days 6–8; Fig 2A) while one patient had negative 16S rDNA (detected but below cut-off) on days 6–8. All four patients with persistent DNAemia at days 13–15 had complicated *S*.*aureus* infections or severe illness (two patients with infective endocarditis, one patient with pancreatic cancer and a catheter-related infection and one with an iliopsoas abscess).

### 16S rDNA load on days 1–2 in relation to sepsis and mortality

**Fig 3:**
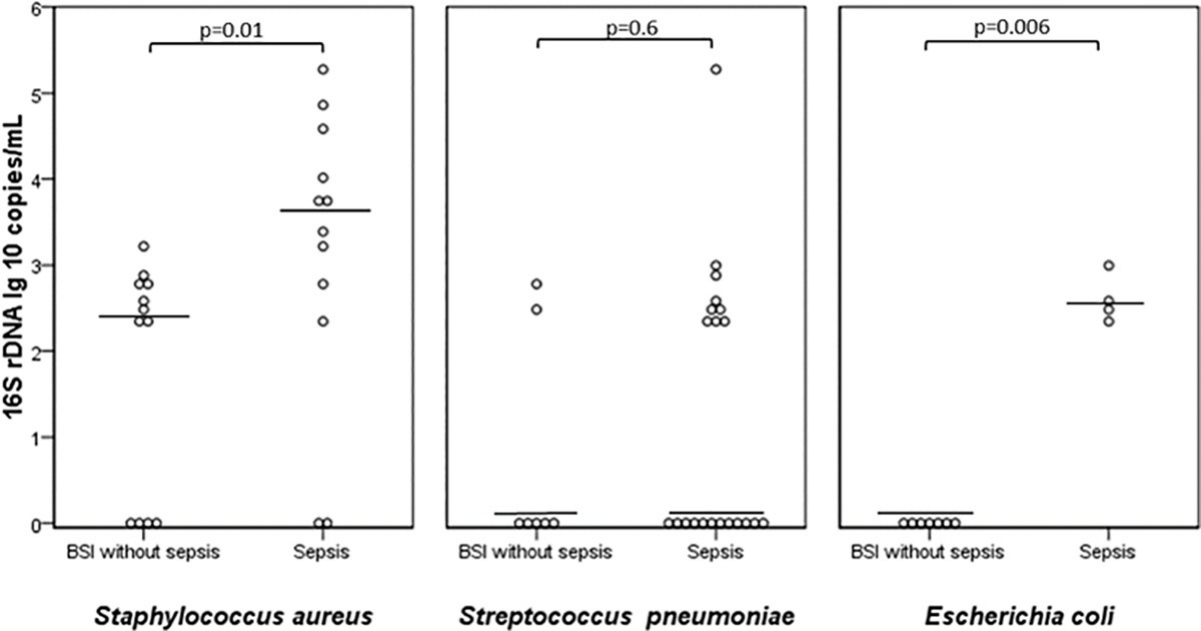
Bacterial DNA load measured by 16S rDNA droplet digital PCR on days 1–2 in patients with bloodstream infection. Comparison between patients with and without sepsis.

BSI patients with sepsis at admission were compared with BSI patients without sepsis for 16S rDNA load at days 1–2 (just after blood culture positivity). Measured in lg10 copies/mL, patients with sepsis had significantly higher 16S rDNA load in blood (median 2.38; range: 0–5.28) than non-septic patients (median 0; range: 0–3.19) (*p* = 0.031). Fig 3 shows the bacterial DNA load on days 1–2 measured by 16S rDNA ddPCR in BSI patients with and without sepsis.

Patients with *S*. *aureus* and *E*. *coli* BSI with sepsis had significantly higher 16S rDNA loads than patients without sepsis, although no such association was found among *S*. *pneumoniae* BSI patients. Patients who died due to infection within 90 days were compared with survivors, and found to have significantly higher 16S rDNA load on days 1–2 in the entire study-group (median 2.83, range: 0–4.58 vs. median 0, range,:0–5.28 lg10 copies/mL) (*p* = 0.006). However, when studied separately, the associations did not reach significance level in any of the different bacteria groups.

Five patients had 16S rDNA loads above 10.000 copies/L on days 1–2 (one patient with *S*. *pneumoniae* BSI with meningitis, three with *S*. *aureus* BSI with endocarditis, and one with prosthetic joint infection).

## Discussion

In this study we developed a ddPCR for quantification of 16S rDNA in whole blood and validated it against species-specific (*nuc*, *lytA*, and *uidA*) ddPCRs by analyzing bacterial-spiked blood samples in 10-fold dilutions and blood samples from patients with *S*. *aureus*, *S*. *pneumoniae*, and *E*. *coli* BSI. The results show that 16S rDNA ddPCR generated higher copy numbers, which can be explained by 16S rDNA’s presence in multiple copies in each bacterial genome. The ddPCR results were concordant between 16S rDNA and species-specific ddPCR in bacterial-spiked samples, and, for *S*. *aureus* and *S*. *pneumoniae*, in BSI patient samples. The inadequate concordance between 16S rDNA and *uidA* DNA from *E*. *coli* BSI patients might be due to few positive samples in the cohort, whereof many had low DNA loads (Fig 1B).

Several previous studies have compared ddPCR with real-time PCR and reported a smaller effect of inhibitors, greater precision, and higher reproducibility [22, 33]. ddPCR has been used for DNA quantification in various fields, such as quantifying HIV viral load [33]. The method has been used only rarely to quantify bacterial DNA in human samples [34] and no previous reports have evaluated this method in BSI assessment.

In virology, the measurement of viral load in blood is well established and used as an important part of diagnostics and treatment-response monitoring in HIV and many other viral infections [35]. Although quantification of pathogenic DNA in BSI assessment has been proposed as a potential prognostic marker [36], research into its role has been sparse. Studies have shown that a high initial DNA load is associated with both sepsis at admission and mortality [7, 10, 12] and that default DNA clearance is associated with treatment failure and mortality [12–14]. Our study found a high initial 16S rDNA load associated with sepsis and mortality, and the few patients with very high DNA loads had severe and complicated infections.

The potential value of pathogen quantification, together with a trend towards broad-range molecular approaches, often targeting rDNA, highlights the importance of correctly interpreting quantitative data from amplified rDNA products. In our study, the concordance between 16S rDNA and species-specific ddPCR was high (Fig 1) without adjusting for species-variation in copy-numbers/bacterial cell. Accordingly, quantitative 16S rDNA ddPCR appears to be a useful marker for bacterial load in BSI patients. However, to determine the true copy number, it is necessary to enable copy number adjustment by identification of the bacteria. Certainly, high-throughput technologies for broad-range species identification is on the march, but in current circumstances a 16S rDNA ddPCR without bacterial identification is considerably more time- and cost-effective. However, knowledge about the etiology is needed for clinical interpretation of the 16S rDNA load, since our results show considerable differences in the dynamics of the DNA load between species (Fig 2). The groups with *S*. *pneumoniae* and *E*. *coli* BSI had a relatively low initial positivity rate, and only a few cases were still 16S rDNA positive on days 3–4. Probably *E*. *coli* and *S*. *pneumoniae* DNA persistence is uncommon in BSI, given that the infections are rightly treated. Accordingly, persistent DNA positivity might be an indicator for treatment failure in BSI of these etiologies.

The group with *S*. *aureus* BSI had a high initial 16S rDNA positivity rate, most cases were still positive on days 3–4, and even on the last sampling point, cases with positivity were noted. (Table 2). BSI persistence is a well-known feature of *S*. *aureus* BSI, linked to complicated infections [37]. This is why routine follow-up BCs are recommended for *S*. *aureus* BSI [38]. According to our results, the value of monitoring bacterial DNA during BSI is probably the greatest in *S*. *aureus* BSI.

In the present study, results in patients with low loads of bacterial DNA showed more discrepancy between 16S rDNA and species-specific DNA. This can be explained by a diminishing performance of the methods in cases of low concentrations of target DNA. Many of the clinical samples were ddPCR-negative even at the first time point. Studies have shown that 50% of BSI episodes have bacterial bloodstream concentrations of only 0.01–1.0 CFU/mL [2, 39, 40]. Even though the pathogen DNA load is reported to be higher [41], the bacterial concentration in BSI is probably frequently below the limit of detection for most molecular methods, especially when small sample volumes of 1–2 mL are used.

Our study has limitations. First, we only included patients with *S*. *aureus*, *S*. *pneumoniae*, and *E*.*coli* BSI, and thus, extrapolation of the results to BSI of all etiologies is precarious. Second, we did not perform any species identification apart from the three species-specific PCR analyzes. Accordingly, there is a possibility that a fraction of the found 16S rDNA derives from other bacteria, possibly due to contamination, poly-microbial or secondary infection. Third, the number of patients in each subgroup with different bacterial etiologies was small, especially in the *S*. *pneumoniae* and *E*. *coli* groups, where samples were frequently ddPCR negative. Finally, not all patients had full sample series. Still, the study provided useful information on the relation between 16S rDNA and species-specific DNA and on dynamic variations between the different bacterial etiologies of BSI.

In conclusion, our results suggest that 16S rDNA ddPCR can serve as a method for broad-range quantification of bacterial DNA load during BSI, with a sensitivity comparable to species-specific ddPCRs. We found that a high initial 16S rDNA load was associated with both sepsis at admission and mortality, which indicates a potential clinical value. Further studies are needed to identify the role of 16S rDNA ddPCR within sepsis management.

## Supporting information

S1 Fig Excel-file including all ddPCR results and clinical data used in the statistical analyses of the study. (XLSX)
